# Applications of Marine Organism-Derived Polydeoxyribonucleotide: Its Potential in Biomedical Engineering

**DOI:** 10.3390/md19060296

**Published:** 2021-05-22

**Authors:** Tae-Hee Kim, Seong-Yeong Heo, Gun-Woo Oh, Soo-Jin Heo, Won-Kyo Jung

**Affiliations:** 1 Department of Biomedical Engineering and New-Senior Healthcare Innovation Center (BK21 Plus), Pukyong National University, Busan 48513, Korea; taehee94@pukyong.ac.kr; 2 Research Center for Marine Integrated Bionics Technology, Pukyong National University, Busan 48513, Korea; syheo@pukyong.ac.kr (S.-Y.H.); ogwchobo@pukyong.ac.kr (G.-W.O.); 3 Jeju Marine Research Center, Korea Institute of Ocean Science & Technology (KIOST), Jeju 63349, Korea; 4 Department of Marine Biology, Korea University of Science and Technology, Deajeon 34113, Korea

**Keywords:** marine organisms, polydeoxyribonucleotide, biomedical engineering

## Abstract

Polydeoxyribonucleotides (PDRNs) are a family of DNA-derived drugs with a molecular weight ranging from 50 to 1500 kDa, which are mainly extracted from the sperm cells of salmon trout or chum salmon. Many pre-clinical and clinical studies have demonstrated the wound healing and anti-inflammatory properties of PDRN, which are mediated by the activation of adenosine A_2A_ receptor and salvage pathways, in addition to promoting osteoblast activity, collagen synthesis, and angiogenesis. In fact, PDRN is already marketed due to its therapeutic properties against various wound healing- and inflammation-related diseases. Therefore, this review assessed the most recent trends in marine organism-derived PDRN using the Google Scholar search engine. Further, we summarized the current applications and pharmacological properties of PDRN to serve as a reference for the development of novel PDRN-based technologies.

## 1. Introduction

Polydeoxyribonucleotide (PDRN), an active mixture of polynucleotides with a molecular weight ranging from 50 to 1500 kDa, is mainly extracted and purified from the sperm cells of Salmon trout *(Oncorhynchus mykiss*) or Chum Salmon *(Oncorhynchus keta*). Importantly, PDRN exhibits a high DNA percentage without active proteins or peptides [1,2]. This compound is currently used as a type of DNA-derived commercial drug, which has been approved by the Korea Food and Drug Administration and is often implemented in tissue repair and wound treatment [3]. PDRN is also reportedly an agonist against the adenosine A_2A_ receptor (A_2A_R), which regulates myocardial blood flow, suppresses immune cell activation, and regulates the release of glutamate and dopamine [4,5]. Additionally, PDRN stimulates nucleic acid synthesis by serving as a source of pyrimidines and purines [5]. Further, PDRN possesses several therapeutic properties, including angiogenesis-promoting, anti-apoptotic, anti-inflammatory, anti-ischemic, and tissue repair activities [6,7,8,9].

**Figure 1:**
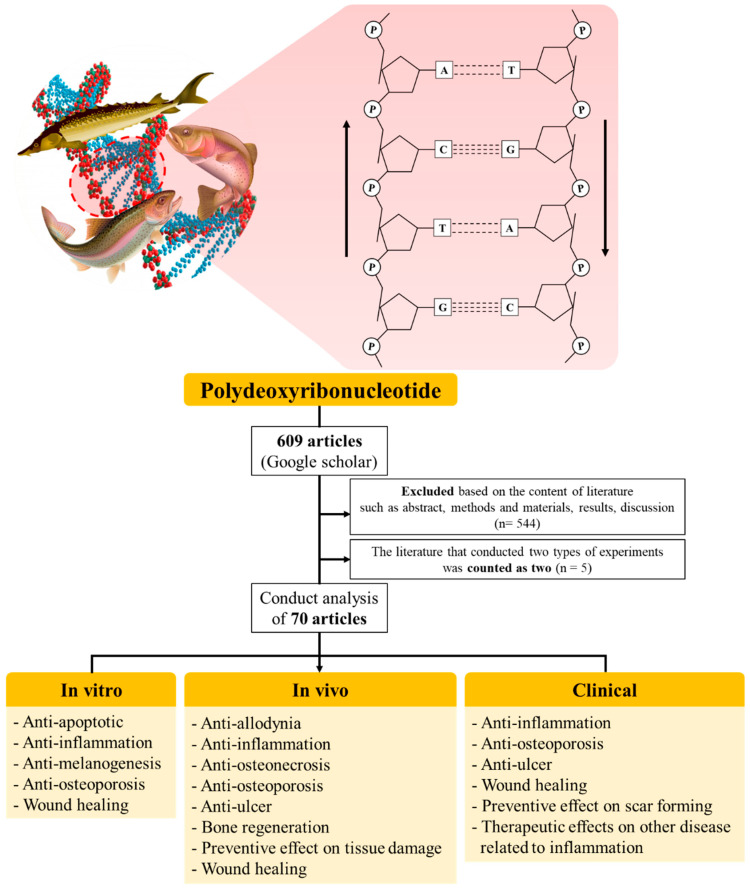
A wide range of therapeutic applications in the field of PDRN.

This review summarizes the current state of PDRN research, including the therapeutic effects and applications of this compound. To facilitate their discussion, these breakthroughs will be classified as in vitro, in vivo, and clinical studies. Finally, we will discuss the challenges and recent trends in PDRN research (Figure 1).

## 2. Results

### 2.1. PDRN-Related Publication Outputs by Year

**Figure 2:**
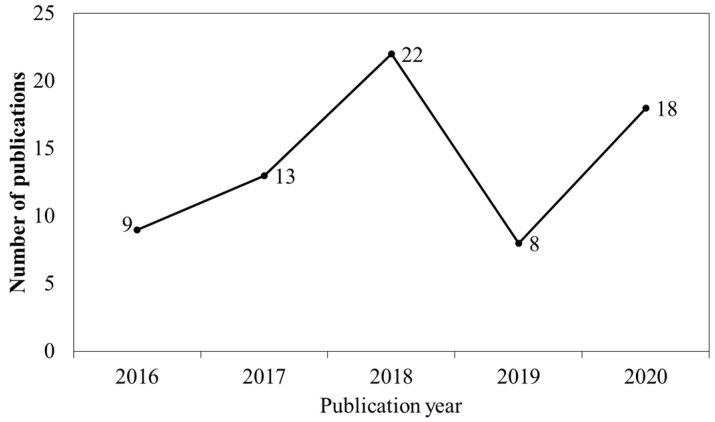
The annual number of articles about the biological effect of PDRN from 2016 to 2020 (n = 70).

Figure 2 illustrates the number of PDRN-related studies published each year. Here, we found that the number of studies on the biological activities of PDRN increased from 2016 to 2020. Intriguingly, 2019 exhibited the lowest publication output in the study period, with only 8 related studies published in this year. However, the number of publications increased rapidly in 2020 compared with 2019. These increasing trends in the research output of PDRN, a novel and safe treatment against several diseases, indicated that interest in characterizing the therapeutic efficacy of PDRN is on the rise.

### 2.2. Source of PDRN Extraction

**Figure 3:**
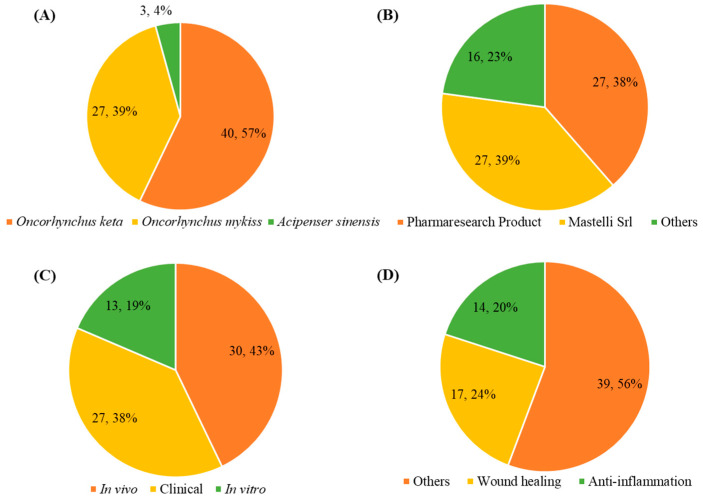
The number of articles about PDRN from 2016 to 2020 (n = 70). (**A**) Extraction sources, (**B**) Companies provided, (**C**) kind of study, and (**D**) pharmaceutical activities of PDRN.

A total of 70 studies were assessed to identify the prevailing sources of PDRN extraction (Figure 3A), from which three sources were identified. PDRN was mainly extracted from *O. mykiss* (40 studies) and *O. keta* (29 studies). Pacific salmon (*O. keta*) contains bioactive compounds that have anti-apoptosis, anti-hypertensive, and bone regenerative properties and is one of the PDRN extraction source used by Mastelli Srl in Italy [10]. Additionally, PDRN is commonly extracted from rainbow trout fish (*O. mykiss*), one of the valuable species processed into skinless fillets in the food industry in South Korea, in order to gain economic competitiveness through substitution of PDRN extracted from *O. keta* (Om-PDRN) [11]. Furthermore, only 3 studies used PDRN extracted from *Acipenser sinensis* (As-PDRN). Sturgeons (*A. sinensis*), a species with the fastest growth rate among the three sources, is a novel PDRN extraction source used by Veritas in Italy [12,13,14,15]. Pharmaresearch Product (Pangyo, Korea), Mastelli Srl (San Remo, Italy), and Veritas (Brescia, Italy) exclusively provide Om-PDRN, PDRN extracted from *O. keta* (Ok-PDRN), and As-PDRN (Figure 3B). Moreover, several companies provide Om-PDRN, including Maruha Nichiro (Tokyo, Japan), BMI Korea (Seoul, Korea), Genoss (Suwon, Korea), BR Pharm (Gangwon-do, Korea), Han Wha Pharma Co., Ltd. (Seoul, Korea), and Kyongbo Pharm (Seoul, Korea).

### 2.3. Therapeutic Effects of PDRN

As mentioned above, the experiments discussed herein will be classified as in vitro studies, in vivo studies, and clinical studies. Most experiments were conducted using in vivo models (30 studies), followed closely by clinical study (27 study), suggesting that in vitro studies are rarely conducted (Figure 3C). Furthermore, the main pharmaceutical properties of PDRN that have been studied are its wound healing (17 studies) and anti-inflammatory (14 studies) effects (Figure 3D). The other therapeutic effects of PDRN are summarized in Table 1. Additional efforts are required to characterize the biological activities and underlying mechanisms of PDRN. The first part of this review explores the biological effects of PDRN from an in vitro standpoint. Particularly, this section will largely focus on the anti-apoptotic, anti-inflammation, anti-melanogenic, anti-osteoporosis, and wound healing effects of PDRN, as well as its other therapeutic properties. In the second part, we will discuss the biological effects of PDRN based on in vivo studies. This section will further address the therapeutic properties of PDRN, including anti-allodynia, anti-inflammation, anti-osteonecrosis, anti-osteoporosis, anti-ulcer, bone regeneration, tissue damage prevention, therapeutic effects on tendon tear, acute tissue injury, and wound healing. The third section will address the biological effects of PDRN determined by clinical studies. Here, we will discuss the clinical properties and applications of PDRN, including its use as an anti-inflammation, anti-osteoporosis, anti-ulcer, wound healing, and scar prevention agent, as well as its potential use in adjuvant therapy, in addition to other therapeutic properties.

#### 2.3.1. Characterization of PDRN Bioactivities Based on In Vitro Studies

One study revealed that Ok-PDRN has anti-apoptotic and anti-inflammatory effects in human bronchial cells stimulated with 10-μm-sized particulate matter (PM) (fine dust) [16]. Ok-PDRN (0–8 μg/mL) ameliorated PM10-induced apoptosis without cytotoxicity on human bronchial cells and suppressed the production of inflammatory cytokines [interleukin (IL)-1β, IL-6, and tumor necrosis factor-α (TNF-α)] and apoptotic factors (caspase-3 and -9). Furthermore, Ok-PDRN induced the activation of cyclic adenosine monophosphate (cAMP), which is activated by A_2A_R, and increased the Bax/Bcl-2 ratio, resulting in anti-apoptosis signal translocation. These results highlighted the potential applicability of Ok-PDRN as a therapeutic candidate for lung damage-associated diseases.

Other studies have demonstrated that Om- and Ok-PDRNs exhibit anti-inflammatory activity on several cell lines [17,18,19]. Ok-PDRN displayed anti-inflammatory activity in a human chondrosarcoma cell line stimulated by IL-1β (10 ng/mL), as well as RAW 264.7 cells stimulated by a combination of zoledronic acid (ZA, 10 μM) and LPS (0.1 μg/mL). These results indicated that Ok-PDRN inhibited the inflammatory reaction by suppressing inflammatory cytokine expression (IL-1β and IL-6) via stimulation of A_2A_R [17,18]. Moreover, 100 µg/mL of Om-PDRN suppressed nitric oxide (NO) production and secretion of pro-inflammatory cytokines (IL-12 and TNF-α) and promoted the production of IL-10, an anti-inflammatory cytokine, in RAW 264.7 cells challenged with *Escherichia coli* lipopolysaccharide (LPS; 10 ng/mL) [19].

Another study investigated the anti-melanogenic effect of Ok- and Om-PDRNs [20]. Both compounds dose-dependently reduced melanin synthesis and tyrosinase activity and 200 μg/mL of both PDRNs resulted in a similar inhibitory effect to that of N’-phenylthiourea (5 μM), which was used as a positive control. Two PDRNs inhibited the activation of microphthalmia-associated transcription factor (MITF), tyrosinase, and tyrosinase-related protein (TRP)-1 by increasing the phosphorylation of extracellular signal-regulated kinases (ERK) and AKT.

Ok-PDRN decreased reactive oxygen species levels and expression of inflammatory mediators and cytokines [cyclooxygenase-2 (COX-2), IL-1β, inducible NO synthase, and TNF-α] on a human chondrocyte cell line treated with 2 mM H_2_O_2_, suggesting the potential applicability of Ok-PDRN as an osteoarthritis (OA) treatment [21]. Furthermore, the authors confirmed that the anti-osteoporosis effect of Ok-PDRN on CHON-001 is mediated by the suppression of ERK and nuclear factor kappa-light-chain-enhancer of activated B cell (NF-κB) phosphorylation. Another study confirmed that Om-PDRN also exhibited an anti-osteoporosis effect on chondrosarcoma cells stimulated with 10 ng/mL of IL-1β [22]. Om-PDRN increased the expression of pro-angiogenic factors, including angiopoietin-2 (ANG-2), platelet-derived growth factor (PDGF), vascular endothelial growth factor (VEGF), and ameliorated the expression of anti-angiogenic factors (endostatin and angiostatin). These results indicated that both Ok- and Om-PDRNs may be used as therapeutic agents to treat OA.

Another study assessed the effects of Ok-PDRN on aging skin cell homeostasis using murine melanoma and human fibroblast [23]. Ok-PDRN reduced the mRNA and protein expression of melanogenic factors (MITF, TRP1, TRP2, and tyrosinase) and tyrosinase enzyme activity, which was consistent with a previous study [20]. Additionally, Ok-PDRN possesses antioxidant properties, as demonstrated by DPPH radical scavenging assay, and induces mitochondrial biogenesis.

Furthermore, co-administration of Ok-PDRN and pirfenidone was found to alleviate the effects of acute respiratory distress syndrome in human lung epithelial cells, which was induced using a combination of LPS (1 μg/mL) and tissue growth factor-β (TGF-β) (5 ng/mL) [24]. This study demonstrated that PDRN inhibited the expression of basic fibroblast growth factor (FGF), collagen (COL) I, connective TGF, and hydroxyproline. Moreover, co-administration of Ok-PDRN with pirfenidone suppressed the expression of inflammatory cytokines (IL-6 and TNF-α). Collectively, the findings of this study suggested that this combined therapy could be used as a new therapeutic approach to treat refractory urogenital ulcers.

Efforts have been made to assess the effects of Ok-PDRN on human neuronal cells for the treatment of postoperative cognitive dysfunction, which is among the most common complications of brain surgery [25]. The sequels are characterized by acute cognitive dysfunction, memory impairment, and loss of attention. Moreover, Ok-PDRN significantly inhibited LPS-induced production and expression of inflammatory cytokines (IL-1β, IL-6, and TNF-α) by suppressing the phosphorylation of cAMP response element-binding protein (CREB), which is mediated by A_2A_R activation. These findings highlighted the excellent therapeutic effect of Ok-PDRN on human neuronal cells challenged with LPS to mimic the symptoms of postoperative cognitive dysfunction.

The wound healing properties of Ok-PDRN have also been assessed using several cell lines [8,26,27,28]. Ok-PDRN significantly increased the cell migration ability on all cell lines, including osteosarcoma cells, CDD-686sk, normal and diabetic human dermal fibroblasts (HDFs), and human umbilical vein endothelial cells (HUVEC). Moreover, Ok-PDRN promoted the activation of the mitogen-activated protein kinase (MAPK) pathway on CDD-686sk cells and increased the expression of VEGF and cluster of differentiation (CD) 31 on normal HGFs. These results suggest PDRN’s potential as a therapeutic agent for bone and skin healing, as well as diabetic wounds.

#### 2.3.2. Characterization of PDRN Based on In Vivo Studies

Four studies assessed the potential of Ok- and Om-PDRNs as anti-inflammatory agents to treat inflammatory diseases in several models [1,2,6,29]. In 2020, one of these studies investigated whether Ok-PDRN exhibited anti-inflammatory activity using Sprague Dawley (SD) rats with ischemic colitis, which was induced via selective devascularization of the descending colon [29]. Ok-PDRN was intraperitoneally injected for 21 consecutive days. The study found that the Ok-PDRN injection ameliorated the increases in skin temperature and mucosal damage induced by ischemic colitis, in addition to suppressing COL deposition in the operated site. Further, protein expression analyses indicated that intraperitoneal (i.p.) Ok-PDRN injection enhanced expression of A_2A_R, VEGF, and caspase-3, as well as of ERK phosphorylation, but reduced the expression ratio of Bax/Bcl-2 and expression of inflammatory proteins, including COX-2, IL-1β, IL-6, and TNF-α. In 2016, two studies confirmed the anti-inflammatory effect of Om-PDRN in combination with hyaluronic acid (HA) using SD rats with induced colitis (i.e., an inflammatory bowel disease of the gastrointestinal tract) [1,2]. Intra-colonic instillation of dinitrobenzenesulfonic acid (DNBS, 25 mg) and oral administration of dextran sulfate sodium (DSS, 8%) successfully induced experimental colitis in SD rats. In both colitis models, Om-PDRN restored normal animal conditions (i.e., colon length, food intake, and body weight) to levels similar to those of the control groups and decreased the colitis-induced macroscopic damage score on both models. Further, colitis induced lipid peroxidation, neutrophil infiltration, and expression of pro-inflammatory cytokines (IL-1β and TNF-α) decreased in both models [1]. However, Om-PDRN treatment exhibited an inhibitory effect on colitis-induced changes [2]. Another study assessed whether Om-PDRN had anti-apoptotic and anti-inflammatory activities on SD rats with LPS-induced lung injuries [6]. Concretely, these injuries were induced via intratracheal LPS instillation (5 mg/kg) and were accompanied by DNA fragmentation. Histological analysis indicated that Om-PDRN administration (8 mg/kg) decreased lung injury score and suppressed DNA fragmentation. I.p. injection of Om-PDRN inhibited apoptotic cell death and inflammatory response by suppressing the expression of IL-6 and TNF-α and decreasing the expression ratio of Bax/Bcl-2. Moreover, the Om-PDRN injection also activated the expression of A_2A_R. Taken together, these results confirmed the potential applicability of Om-PDRN as a novel treatment for lung injury due to its anti-apoptotic and anti-inflammatory properties.

Allodynia results from nerve damage or dysfunction of the somatosensory system and severely reduces the quality of life of patients, as innocuous stimuli such as the touch of clothing cause them continuous pain and discomfort [30]. Several inflammatory agents have been found to induce astrocyte activation, which promotes the development of allodynia [31,32]. Therefore, Lee, S.H., et al. (2020) investigated whether Om-PDRN could alleviate allodynia via its anti-inflammatory effect using neuropathic pain-induced models [33]. Notably, Om-PDRN (20 mg) injection at pain site alleviated mechanical allodynia and reduced glial fibrillary acidic protein (GFAP) expression, which is predominantly associated with neuro-inflammatory states, on spinal nerve ligation (SNL)- and chronic post-ischemia-induced pain models. Therefore, this study confirmed the potential use of Om-PDRN as a neuropathic pain treatment.

Previous studies have confirmed that Ok- and Om-PDRNs prevent osteonecrosis induced by physical and chemical factors, including administration of bisphosphonate, intra-articular injection of monosodium iodoacetate (MIA), and surgery on the anterior cruciate ligament (ACL) [7,21,34]. In the ZA-induced osteonecrosis model, injection of Ok-PDRN into soft tissues near the defect sites decreased the severity of osteonecrosis, necrotic bone formation, and detached osteoclasts [7]. Compared to the osteonecrosis-induced group, Ok-PDRN injection improved the number of blood vessels, attached osteoclast, and enhanced bone remodeling in the sockets. Furthermore, another study also indicated that oral administration of Ok-PDRN inhibited the expression of IL-1β, matrix metalloproteinase (MMP)-3, and MMP-7, as well as production of inflammatory mediators including COX-2, prostaglandin E_2_ (PGE_2_), and TNF-α in an MIA-induced osteonecrosis model [21]. Furthermore, upregulating inflammatory cytokines and MMPs reportedly induced chondrocyte apoptosis and cartilage degradation. Additionally, Ok-PDRN ameliorated osteonecrosis by suppressing ERK phosphorylation. These results suggest the potential applicability of Ok-PDRN as a therapeutic agent for OA. Intra-articular injection of Om-PDRN also exerted chondroprotective effects in an experimentally induced OA model [34]. Om-PDRN significantly attenuated total Mankin scores, which are used to evaluate the OA pathological changes, including structural integrity, and cellular changes.

A 2018 study reported that Ok-PDRN, both alone and in combination with pantoprazole (i.e., a proton pump inhibitor), alleviated the symptoms of gastric ulcers (GU) [35]. Further, both Ok-PDRN treatments improved tissue regeneration and decreased ulcer size in an indomethacin (IND)-induced GU model. Additionally, molecular analysis indicated that Ok-PDRN suppressed the expression of pro-inflammatory cytokines, including IL-6, IL-1β, and TNF-α, and increased the expression of VEGF and A_2A_R via activation of the cAMP-protein kinase A (PKA) pathway. Therefore, the findings of this study suggest that both Ok-PDRN and pantoprazole co-administration and Ok-PDRN monotherapy rendered more potent therapeutic effects against GU than pantoprazole therapy alone.

Two studies confirmed that Om- and As-PDRNs regenerated bone tissue in combination with subcutaneous implants using calvarial defect models [13,36]. Concretely, the implantation of Om-PDRN combined with a human demineralized dentin matrix in a subcutaneous pouch promoted osteoinduction in a nude mice model in one of the aforementioned studies [36]. Histological analyses elucidated a fibrous capsule near the implant material, as well as several dentin particles, deposition and calcification of new bone (NB) matrix, and an increase in the number of bone-forming cells and blood vessels. Additionally, this therapy expanded the area of NB growth on the surface of dentin particles and increased the number of cells (osteoblasts and fibroblasts) attached to the surface of the implant material. Moreover, the other study demonstrated the bone regenerative effects of As-PDRN using a calvarial defect rat model [13]. Filling the defect site with As-PDRN significantly increased the percentage area of NB and production of osteocalcin (OCN) and osteopontin (OPN).

Oral administration of Om-PDRN (8 mg/kg) also exerted a mucoprotective effect on IND-induced gastropathy [37]. Om-PDRN favored mucosal tissue regeneration by decreasing histological alterations including mucosal injury, loss of epithelial layers, and distortion of the mucosa and mucosal glands. Further, Om-PDRN treatment decreased ulcer indices and tissue damage score. Molecular analyses revealed that Om-PDRN suppressed the expression of pro-inflammatory cytokines (IL-1β, IL-6, and TNF-α) and decreased the Bax/Bcl-2 expression ratio via MAPK activation. cAMP expression was also induced by Om-PDRN in the gastric tissue and serum. Therefore, these results demonstrated the mucoprotective effect of Om-PDRN via activation of A_2A_R.

I.p. administration of As-PDRN (8 mg/kg/day) has also been reported to render neuroprotective effects [14]. For instance, protein expression analyses indicated that As-PDRN treatment suppressed malondialdehyde (MDA) and mTOR levels in the brain, while also increasing glutathione (GHS) and brain-derived neurotrophic factor (BDNF) levels. Moreover, As-PDRN attenuated brain edema, ameliorated neuronal morphological changes such as neuronal loss, number of degenerating pyramidal cells, and interstitial edema, and enhanced escape latency time on a brain damaged mice model. In conclusion, As-PDRN has a protective effect against cadmium (Cd)-induced brain injury.

A 2017 study confirmed that Om-PDRN prevents tissue damage in C57 BL/6J mice [38]. Concretely, the mice were challenged with Cd to induce the blood-testis barrier (BTB), resulting in a combination of tight junctions, adherent junctions, and gap junctions. Om-PDRN was administered immediately after Cd exposure. Om-PDRN treatment suppressed the phosphorylation of ERK and decreased follicle-stimulating hormone (FSH) and luteinizing hormone (LH) levels. Additionally, Om-PDRN treatment increased the size of seminiferous tubules, enhanced Johnsen’s score, and increased testosterone (TE) and inhibin B serum concentration. These results were consistent with histological analyses, which indicated a decrease in the isolation of peripheral positive germ cells and macrophages in the extratubular compartment, as well as a downregulation of TGF-β3 and integral membrane proteins (Claudin-11, occludin, and N-Cadherin). These results demonstrated the ability of Om-PDRN to restore Cd-induced injury on the BTB, thereby improving Cd-inhibited spermatogenesis.

In 2020, three studies assessed the applicability of Ok- and Om-PDRNs as preventive treatments against acute tissue injury [4,39,40]. One of these studies conducted single i.p. Ok-PDRN injections on an acute lung injury model, which was induced via LPS intratracheal instillation [39]. A reduction in lung injury scores was observed 12 h after injection, resulting in the occurrence of patch intra-alveolar macrophages, normal-looking alveolar structures, and a reduction in inflammatory cell infiltration. Om-PDRN also decreased the number of white blood cells and bronchoalveolar lavage fluid (BALF) cells. Further, molecular analyses indicated that Om-PDRN injection suppressed the expression of inflammatory cytokine (IL-1β, IL-6, and TNF-α) in the BALF, serum, and lung tissue, activated the NF-κB and MAPK pathways, and phosphorylated CREB and PKA. Moreover, Om-PDRN inhibited cell apoptosis by suppressing the expression of cAMP in BALF, decreasing the Bax/Bacl-2 expression ratio, and inhibiting histological expression of cleaved caspase-3 and cleaved caspase-9. Another study confirmed that Ok-PDRN provided protection against acute tissue injury in a CCl_4_-induced injury model [4]. A single i.p. injection of Ok-PDRN (8 mg/kg) was administered once a day for seven days after the induction of acute liver injury via CCl_4_ i.p. injection. Morphological and histological analyses indicated that the Ok-PDRN injection suppressed gross morphological alteration of the liver, including scattered white granules, graying, brittle texture, and liver enlargement, and decreased liver weight, liver index, and liver histopathological score, which are characteristically increased by CCl_4_-incudced liver injury. Molecular analyses indicated that this therapy inhibited oxidative stress, immune cell activation, and apoptosis resulting from CYP2E1 and UCP2 downregulation in the liver, suppressed the inflammatory cytokine profiles (IL-1, IL-6, and TNF-α) in serum and liver tissue, inhibited cleaved caspase-3 and cleaved caspase-9 expression, and reduced the Bax/Bcl-2 expression ratio. Additionally, this study confirmed that the tissue damage preventive effects of Ok-PDRN were mediated by the regulation of NF-κB and MAPK activation. Another study assessed the ability of Om-PDRN to prevent acute tissue injury [40]. In a similar manner to the above-described study, acute liver injury was induced by an i.p. CCl_4_ injection, after which Om-PDRN injections were administered for seven days [4]. Om-PDRN decreased serum aspartate aminotransferase (AST) and alanine aminotransferase (ALT) concentrations and inhibited inflammatory cytokine expression (IL-1β, IL-6, and TNF-α). Moreover, this compound activated A_2A_R expression and reduced the Bax/Bcl-2 expression ratio, as well as the percentage of terminal deoxynucleotidyl transferase dUTP nick end labeling (TUNEL)-positive cells. In summary, these studies demonstrated that Ok- and Om-PDRNs prevented acute liver injury in several models, thus demonstrating the applicability of these compounds for the treatment of acute liver injuries.

Five studies have also suggested that Ok- and Om-PDRNs can favor the regeneration of torn tendons [5,41,42,43,44]. A combined treatment consisting of Ok-PDRN and umbilical cord blood-derived mesenchymal stem cells (UCB-MSCs) coupled with microcurrent therapy also exhibited regenerative effects on a rotator cuff tendon tear (RCTT) model [41,42]. Ok-PDRN injection coupled with microcurrent therapy reduced the mean tendon tear size and increased the number of VEGF-positive cells and platelet endothelial cell adhesion molecule (PECAM)-1-positive microvascular densities. Motion evaluation prior to injection and 4-weeks post-injection suggested that this therapy lengthens the walking distance and increases fast walking time. Another study by the same author confirmed that Ok-PDRN injection combined with UCB-MSCs treatment rendered the same therapeutic effect as Om-PDRN [5,42]. This combined therapy decreased subscapularis tendon tear size (STTS) and promoted regeneration of new COL fibers, resulting from increasing Masson’s trichrome-stained fiber and COL 1-positive cell densities. Additionally, the authors observed extensive proliferating cell nuclear antigen staining in the regenerated COL fibers and enhanced platelet PECAM-positive microvascular density. Further, function evaluation using motion analysis showed that Ok-PDRN injection coupled with UCB-MSCs lengthens walking distance and increases fast walking time and walking speed.

Direct Om-PDRN administration and i.p. injections at the wound site promoted the regeneration of the injured tendon on an RCTT model and an Achilles tendon injury model [5,43,44]. In a study from 2017, i.p. Om-PDRN injections enhanced the cross-sectional area (CSA) of the laceration sites and resistance to mechanical stress and stored energy [43]. Due to the drawbacks of this study, including lack of histological evaluation and determination of underlying molecular mechanisms, another study was conducted to confirm the therapeutic effects of Om-PDRN on an Achilles tendon injury [44]. The tendon tear size of the RCTT model decreased after Om-PDRN injection with UCB-MSCs, and COL fiber regeneration was observed, in addition to numerous proliferating nuclear antigen- and BrdU-stained cells. Moreover, Om-PDRN increased the number of VEGF-positive cells and PECAM-1-positive microvascular densities. In the case of the Achilles tendon injury model, it was confirmed via von Frey filament and plantar tests that an Om-PDRN injection once every 2 days for 16 days encouraged tactile threshold and paw withdrawal latency in a mouse model, indicating the regeneration of torn tendons [41]. Molecular analyses demonstrated that Om-PDRN decreased the production of IL-6 and TNF-α, suppressed the activation of the cAMP-PKA-CREB pathway, and attenuated apoptosis, which resulted from a decrease in the number of cleaved caspase-3- and cleaved caspase-9-positive cells and the Bax/Bcl-2 expression ratio. These results demonstrated the potential applicability of Ok- and Om-PDRNs as effective alternatives for tendon healing in combination with other therapeutic agents.

A total of nine studies have evaluated the wound healing activity of Ok- (six studies) and Om-PDRNs (three studies) [4,8,9,27,28,45,46,47]. In the case of Ok-PDRN, one study has evaluated the effective range of Ok-PDRN for wound healing as a function of molecular weight distributions (under 50 kDa, between 50 and 1500 kDa, over 1500 kDa) [27]. Regardless of MW distribution, daily i.p. injection of PDRNs (8 mg/kg) promoted wound closure and COL production at the wound site and decreased wound area compared to the control group (0.9% NaCl i.p. injection). The authors also observed less lipid accumulation in the PDRN groups and normal wound healing (i.e., not excessive wound healing). A 2019 study hypothesized and confirmed that Ok-PDRN promotes the survival of composite grafts [45]. Ok-PDRN (0.75 mg/kg) was injected evenly into the surrounding skin of the composite graft, whereas normal saline (0.9% NaCl) was used as a negative control. The injection increased the viable area around the composite graft and the number of CD31-stained capillaries, thus indicating neovascularization. The authors also observed that the blood flow signal was initially localized at the margin of the composite grafts, but spread to most of the graft area by day 12. Another study confirmed the therapeutic effect of Ok-PDRN on diabetic wounds using transgenic and diabetic animal models [28]. Full-thickness skin wounds were created on the back of each mouse using a 4 mm diameter biopsy punch, after which an intradermal injection of Ok-PDRN was performed daily into the edges of the wound site. Intradermal Ok-PDRN injection promoted the formation of granulation tissue and capillary blood vessels and enhanced the proliferation of fibroblasts at the diabetic wound site. Histological evaluation revealed that PDRN quickly reduced diabetic wound depth and width, thickened the epidermis with re-epithelialization, and promoted epithelialization of the epidermis around the wound. Another study also reported the regenerative effects of Ok-PDRN on diabetic wounds using a diabetic mouse model with a punch biopsy tool [8]. The authors of this study developed an Ok-PDRN-loaded alginate hydrogel (Alg-PDRN hydrogel) and evaluated its wound-healing effect on the full-thickness wound model. Compared with saline and PDRN injection groups, the Alg-PDRN hydrogel group exhibited a completed re-epithelialization of the wound site, resulting from the increased wound closure rate, granulation tissue thickness, and COL density of the regenerated tissue, coupled with a reduction in the number of inflammatory cells. Additionally, molecular analyses indicated that Alg-PDRN hydrogel implantation at the wound site attenuated inflammatory-associated protein expression [TGF-β and myeloperoxidase (MPO)] while favoring the expression of VEGF and α-smooth muscle actin (α-SMA).

Moreover, two studies have examined the wound healing effect of Om-PDRN using several animal models [9,46]. One of these studies reported that daily i.p. Om-PDRN injection favored the recovery of a model of fractional ablative CO_2_ laser-induced skin resurfacing [46]. However, this treatment had some negative side effects, including prolonged erythema, edema, crusting, and hyperpigmentation [46]. According to histological analyses, Om-PDRN promoted the wound healing process, the degree of epithelial confluence, and improved general wound appearance with less erythema and crusting. Additionally, the granulation tissue thickness score was also increased by Om-PDRN injection. Furthermore, molecular analyses indicated that Om-PDRN treatment increased the number of VEGF-positive cells and PECAM-1/CD31-positive microvessels and enhanced VEGF production. Another study from 2017 demonstrated the potential of Om-PDRN for the prevention of scar formation and wound healing in an incisional wounded rat model [9]. The experimental wounds were made by removing the skin and the panniculus carnosus muscle, after which Om-PDRN was intraperitoneally injected at both three and seven days at wound sites. Om-PDRN treatment not only decreased the number and infiltration of inflammatory cells compared to the sham group but also reduced scar size. Furthermore, the number of COL fibers in the wound area increased after Om-PDRN treatment. Om-PDRN injection also suppressed inflammatory-associated protein expression (high mobility group box-1, HMGB-1) and promoted COL synthesis (COL I and III). Only one study has compared the therapeutic efficacy of Ok- and Om-PDRNs on wound healing [47]. Two PDRNs were intraperitoneally injected every day on each PDRN injection group after the induction of full-thickness skin lesions in mice. These PDRN treatments enhanced the wound healing progress, as well as re-epithelialization and granulation tissue proliferation. Histological analyses indicated that the PDRN injections reduced the infiltration of inflammatory cells, thus demonstrating the anti-inflammatory effect of the two evaluated PDRN treatments on wounds. Furthermore, these therapies upregulated the expression of several wound healing-associated proteins, such as p63, PECAM/CD31, VEGF, and TGF-β. Moreover, no statistically significant difference was observed between the two PDRN groups. Overall, these studies indicated the wound-healing effects of Ok- and Om-PDRNs, both of which displayed a similar therapeutic effect.

#### 2.3.3. Characterization of PDRN Based on Clinical Studies

Many clinical studies have revealed that PDRN from marine organisms prevents numerous inflammation-related diseases, including bursitis, fasciitis, mucositis, and tendinopathy [15,48,49,50,51,52,53]. For instance, Om-PDRN has been found to be an effective treatment against genital lichen sclerosus (LS), supraspinatus and rotator cuff tendinopathy, and pes anserine (PA) bursitis [48,49,50,51]. In one study, twenty-one male patients with genital LS (ages 34 to 77) were examined to investigate the therapeutic effects of loco-regional intradermal (prepuce) or submucosal (corona sulcus/glans) Om-PDRN injections [48]. Based on the dermatology life quality index, Om-PDRN injections for 10 weeks improved the overall quality of patients without adverse reactions and pain. Moreover, Om-PDRN treatment did not significantly affect sexual function according to the international index of erectile function. Another study confirmed the therapeutic potential of ultrasound (US)-guided subacromial bursa Om-PDRN injection as a therapeutic alternative for the treatment of chronic supraspinatus tendinopathy [49]. The shoulder pain and disability index (SPADI) score and visual analog scale (VAS), both of which are widely used as indices of pain, were significantly decreased by Om-PDRN treatment. Additionally, US-guided Om-PDRN prolotherapy also showed potential as a conservative treatment to reduce the pain of rotator cuff tendinopathy pain [50]. For 32 patients with chronic rotator cuff disease (RCD) (from 30 to 75 years of age), weekly Om-PDRN injections (maximum of 5 times) remarkably reduced shoulder pain, as well as the SPADI and VAS scores. Shoulder function was assessed via a single numeric evaluation. The results indicated that Om-PDRN injection improved the shoulder function of patients without observable complications such as infection, allergic reaction, or post-injection pain. Another study investigated the potential applicability of Om-PDRN as a therapeutic agent against PA bursitis, a common soft tissue pain disease that causes medial knee pain [51]. In this study, US-guided PA bursa Om-PDRN injections were administered to a 50-years-old female patient who experienced chronic refractory pain in the inner knee area during daily activities, including climbing stairs and walking. Om-PDRN therapy alleviated pain and reduced the numeric rating scale (NRS) score, which measures the pain intensity of patients, as determined by a 2-week follow-up assessment. Moreover, full recovery was observed after eight months without any side-effects. Therefore, the findings of this study highlight the promising potential of PDRN injections as an efficient therapeutic strategy to treat PA bursa. Two studies have also investigated the potential applicability of Ok- and Om-PDRNs as therapeutic agents for the treatment of inflammatory diseases including lateral epicondylitis and plantar fasciitis [52,53]. The effect of Ok-PDRN on lateral epicondylitis, the most common cause of elbow pain in adults, was evaluated on two patients experiencing right lateral elbow pain exacerbation [52]. Ok-PDRN injection (1 vial, 5.625 mg/3 mL) into the common extensor tendons alleviated pain, as demonstrated by a decrease in NRS and improvement of the hypervascularity at the injected site. More importantly, the symptoms of lateral epicondylitis were successfully ameliorated without any complications.

Additionally, a comparative study was conducted to assess the therapeutic efficiency and safety of Ok-PDRN and triamcinolone (TAC) for the treatment of plantar fasciitis [53]. Ok-PDRN was injected three times at 1-week intervals, whereas TAC was injected only at the first treatment, followed by normal saline on the second and third treatments. Ok-PDRN and TAC injection improved the symptoms of plantar fasciitis, as demonstrated by a decrease in VAS and Manchester-Oxford Foot Questionnaire (MOXFQ) scores (i.e., a patient-reported outcome measure) without any complications. This study demonstrated that PDRN injection continuously relieved pain throughout a 6-month period and its effect on pain intensity was comparable to that of TAC. A 2018 study confirmed the therapeutic effects of As-PDRN on three patients who developed oral mucositis due to radiotherapy [54]. After radiotherapy on the oral cavity, As-PDRN was sprayed twice or three times a day in areas exhibiting mucositis symptoms for at least a month. All patients experienced pain relief approximately 2–3 days after treatment. Moreover, erythema and desquamation reductions were observed within a week and the patients did not report any allergic reaction. Based on these results, the authors of this study concluded that radiotherapy-induced oral mucositis could be effectively treated by spraying the oral cavity with As-PDRN.

In a 2018 study, mucosal Ok-PDRN injections were performed on five patients ranging from 65- to 79-years of age to assess their potential as an osteonecrosis treatment [55]. The patients were fist treated with oral bisphosphonates or methotrexate for disease treatment, after which a medication-related osteonecrosis of the jaw (MRONJ) diagnosis was conducted. In this study, Ok-PDRN was injected daily at the operated site (maxilla and mandible) after surgical debridement. All patients experienced pain relief after two weeks of daily Ok-PDRN injection. Moreover, the surgery area exhibited full soft tissue coverage without any observable signs of infection, demonstrating the applicability of Ok-PDRN injections for MRONJ treatment.

A 2017 study investigated the capacity of Ok-PDRN to inhibit diabetic foot ulcers [56]. In this study, intramuscular Ok-PDRN administration was conducted after initial surgical debridement of the necrotic tissue on type 2 diabetes patients exhibiting diabetic foot ulcers. Ok-PDRN administration for 2 weeks suppressed transcutaneous oxygen tension, ameliorated inflammatory response and neutrophil pigmentation, and promoted the formation of granulation tissue with higher-density vascular markings.

The potential applicability of Ok-PDRN as an adjuvant therapeutic agent for revision rhinoplasty was also assessed [57]. This study conducted intra-lesional Ok-PDRN injections and invasive radiofrequency treatment on thirty 23- to 62-year-old Korean patients with contracted noses. After one week, the combination of Ok-PDRN pretreatment and invasive bipolar radiofrequency therapy significantly softened the skin of contracted noses and improved nasal skin mobility. Additionally, most patients observed moderate clinical improvement in nasal tip dimpling and contracture. Therefore, the authors suggested that PDRN preoperative adjuvant treatment, coupled with bipolar radiofrequency therapy, improved rhinoplasty results without major side effects.

A 2018 study confirmed the preventive effect of Ok-PDRN on post-surgery scar formation [58]. Forty-four patients who underwent open total thyroidectomy were subjected to a combined therapy consisting of fractional laser treatment (48 J/16 cm^2^) and Ok-PDRN injections during the first four weeks after surgery, after which Ok-PDRN injections were administered for an additional 2-week period. After a 16-week follow-up assessment, the treatment outcomes were evaluated using the Vancouver scar scale (VSS) score. Notably, the score of the Ok-PDRN injection group was more than 50% lower than that of the saline injection group. Furthermore, objective therapeutic outcomes were evaluated using three-dimensional image analysis and spectrophotometry. Based on these analyses, Ok-PDRN injection improved pigmentation, vascularity, scar size, and erythema index (EI) without any observable adverse events. Therefore, adjuvant administration of Ok-PDRN after laser treatment is a promising preventive treatment to minimize scar formation after thyroidectomy

Moreover, two studies investigated the effects of Om-PDRN and HA co-administration as a potential treatment for osteoporosis [59,60]. A total of 29 patients experiencing persistent knee joint pain and 98 OA patients were intra-articularly injected with HA alone or a combination of Om-PDRN and HA. In a study published in 2019, Om-PDRN and HA co-administration reduced VAS, knee society scores (KSS), and Western Ontario and McMaster Universities OA index on pain and physical function. Additionally, this treatment did not induce any adverse events, drug reactions, or complications. Similarly, another study administered Om-PDRN injections to 98 patients with knee OA and demonstrated that this treatment also significantly decreased the VAS score of patients after 12 months of treatment. Based on these studies, Om-PDRN and HA co-treatment represents an effective and safe therapeutic alternative for osteoporosis treatment.

The potential use of Ok-PDRN to treat peripheral neuropathy (i.e., peripheral nerve damage) has also been assessed in other studies [61,62,63,64,65]. For instance, a US-guided Ok-PDRN injection was administered to a patient with sudden left upper limb pain and weakness after herpes zoster [61]. The patient experienced pain relief one day after peri-branchial plexus Ok-PDRN injections, as indicated by a decrease on NRS from 9 to 4, after which additional weekly injections were performed for 6 months. Upon completing the treatment, the pain NRS was maintained at 3 and improvements in motor weakness were confirmed using magnetic resonance imaging. Moreover, Ok-PDRN is also considered a promising and side-effect-free corticosteroid alternative for the treatment of carpal tunnel syndrome (CTS) and peripheral neuropathy of the upper extremity associated with the compression of the median nerve [64,65,66]. In two studies, a US-guided Ok-PDRN injection (1 vial, 5.62 mg/3 mL) was administered to treat the carpal tunnel of a patient with type 2 diabetes mellitus (41-years-old) and 30 patients with CTS, respectively [61,62]. After two Ok-PDRN injections, there was a significant improvement in NRS, Boston CTS questionnaire on severity, and Boston CTS questionnaire on function scores. After a 6-month follow-up assessment, the CSA of the median nerve was reduced and CTS symptoms improved without any side effects. Moreover, Ok-PDRN reportedly alleviated the symptoms of hemiplegic shoulder pain caused by subacute stroke [64]. Concretely, twenty hemiplegic patients with limitation of passive external rotation of the shoulder were subjected to intra-articular injections of Ok-PDRN for two weeks. Decreased NAS score and improvements in passive range of motion (PROM) were observed after the aforementioned intra-articular injections. Moreover, although these differences were not statistically significant, the efficiency of the tested treatment was largely similar to that of a TAC injection. The results of these studies suggested that Ok-PDRN injection is a safe alternative to corticosteroid injection for the treatment of several diseases including CTS, hemiplegic shoulder pain, and neuropathic pain.

Two studies investigated the potential use of two PDRNs (Ok- and Om-PDRN) as pain relief agents, respectively [65,66]. One of these studies reported that Ok-PDRN treatment alleviated the radiating leg pain of a 51-year-old man presenting cystic mass lesions on the inner aspect of the right sciatic foramen, which did not improve after a transforaminal epidural steroid injection prior to Ok-PDRN injection [65]. In this study, Ok-PDRN injection was administered three times in the same site at 2-weeks intervals. The pain decreased after the injection and disappeared completely after 2 months. Therefore, this study confirmed that Ok-PDRN injection could be used as an effective means to treat leg pain. The other study assessed the therapeutic effects of Om-PDRN on complex regional pain syndrome (CRPS) type II [66]. A 32-year-old patient presenting left leg numbness after a traumatic event was subjected to prolotherapy using Om-PDRN at the ventral surface of the left L5 transverse process. One day after prolotherapy, the authors observed improvements in allodynia and hyperalgesia coupled with diminished skin flushing. These results indicated that prolotherapy using Om-PDRN alleviated the symptoms of CRPS II. Therefore, both studies confirmed the potential applicability of PDRNs for pain treatment.

Previous studies have reported that 1927-nm fractionated thulium laser energy is a safe alternative for treatment of male or female pattern hair loss (PHL) (i.e., the most common type of alopecia in both men and women) [67]. However, to enhance the therapeutic efficacy of thulium laser energy, two studies combined this therapy with Ok- and Om-PDRN, respectively [68,69]. In the case of the Om-PDRN study, intra-perifollicular injection after 1927-nm fractionated laser energy treatment (Option 1) and intra-perifollicular injection without thulium laser treatment (Option 2) were performed on eight patients aged 26 to 46 [69]. None of the patients had previously taken topical or oral hair loss medications. The results of this experiment indicated that the mean hair thickness and count were greatly improved by combining Om-PDRN and thulium laser therapy compared to Om-PDRN injection only. In the case of the Ok-PDRN study, a 55-year-old male alopecia patient received 12 weekly sessions of Ok-PDRN intra-perifollicular injection after 1927-nm fractionated laser energy treatment. The patient was satisfied with the improvements in hair counts and hair thickness. Furthermore, the patient also observed improvements in hair graying and pigmented hair shaft counts along the frontal hairline and vertex area [68]. Therefore, these studies suggested that both Ok- and Om-PDRN enhance the efficacy of thulium laser therapy for treatment of hair graying and hair loss.

Two studies hypothesized that Om-PDRN could be used as an efficient therapeutic agent for the treatment of posterior tibial tendon dysfunction and tendon tear [70,71]. In 2016, a study administered US-guided injections of Om-PDRN (1 vial, 5.625 mg/3 mL) to a 67-year-old woman experiencing pain along the inside of the foot and ankle at the site of the posterior tibial tendon [70]. Four injections were administered into the tibialis posterior muscle at 1-week intervals. The injections significantly decreased the NRS score and pain without any observable complications. At the final follow-up physical examination, the posterior tibial tendon exhibited no swelling and tenderness and there was a marked improvement in tendon strength. Another study investigated whether US-guided Om-PDRN injection could be used to safely treat a partially torn tendon [71]. Seventeen patients were subjected to US-guided injections into a well-defined and hypoechoic or anechoic area of the supraspinatus tendon for 4 weeks at 2-week intervals. This study assessed the patients for clinical outcomes on weeks 0, 6, and 12. Scoring analyses indicated that the Om-PDRN injections significantly improved arm, shoulder, and hand disability subscore, as well as VAS. A clinical evaluation indicated that Om-PDRN treatment decreased the tear volume of the supraspinatus tendon and increased the active range of motion (ROM) in the forwarding flexion. Additionally, no adverse side effects were observed in any of the study participants except for one. Therefore, this study demonstrated the potential of US-guided Om-PDRN injection as a promising and safe procedure to treat shoulder pain caused by a rotator cuff tear.

Om-PDRN injection has also been used to regenerate traumatic nerve injury. In one study, a 54-year-old patient with traumatic cervical spine fracture was treated with a US-guided injection (5.625 mg/3 mL) at the left C5 nerve root block [72]. The first injection improved the neck and left shoulder pain but did not enhance the motor power of the left upper extremity. However, the second injection, which was administered one week after the first, improved the motor power of the left shoulder elevation and elbow flexion, as well as neck and left shoulder pain. Moreover, Om-PDRN injection into injured nerve roots enhanced the compound motor action potential amplitude of the left musculocutaneous, axillary, and suprascapular nerve, as demonstrated by electrophysiological analyses conduced two months after the injury. Therefore, the authors confirmed that US-guided Om-PDRN injection is a safe and useful treatment for cervical nerve root injury through the alleviation of clinical symptoms, including motor weakness and neuropathic pain.

Wound healing through activation of A_2A_R is among the main pharmacological activities of Om- and Ok-PDRNs. Therefore, three clinical studies have been conducted to prove their wound healing properties [73,74,75]. Ok-PDRN was investigated as a potential therapeutic agent for the treatment of a chronic non-healing wound [73]. Chronic non-healing wounds require especially quick wound treatment to decrease the risk of infection and hospitalization [76]. Before Ok-PDRN injection, a 28-year-old male patient with a third-degree burn was subjected to fasciotomy, irrigation, and debridement to heal chronic non-healing wounds. However, a full regeneration of the stump wound was not achieved with these treatments after 7 months, and therefore an alternative Ok-PDRN-based treatment was tested. Intradermal Ok-PDRN injections coupled with hydrophilic polyurethane foam dressing were applied three times into open wounds on the first, fourth, and ninth days of the experiment. The injections improved the lesions enough to allow the patient to wear the prosthesis after four weeks. These results demonstrate that Ok-PDRN injections can be used as an effective treatment for non-healing wounds without the need for additional therapies.

Restoring the original appearance of an ear after amputation is widely known to be uniquely challenging [77]. However, several adjunctive postoperative treatments have been developed to improve ear reconstruction results, including hyperbaric oxygen therapy (HBOT), as well as the administration of aspirin, prostaglandin, platelet-rich plasma (PRP), and dextran-40 [73]. Recently, however, a study was conducted using Om-PDRN injections along with local HBOT and PRP injections to improve the therapeutic efficacy [73]. An amputated ear was reattached to the original anatomical location of a 76-year-old man experiencing total left ear amputation, and the replanted ear was covered to initiate local continuous HBOT for 2 weeks after replantation. Two days after the operation, to initiate tissue perfusion in the replanted ear, PRP and Om-PDRN injections were administered into the intact ear near the injury site every 3 days (for 9 days) and 2 days (for 10 days), respectively. This combined therapy maintained the survival of the composite ear graft without tissue loss and the ear was almost completely healed after 53 days. In 2020, another study also investigated the therapeutic effects of Om-PDRN on a woman suffering from a disabling post-surgery hypertrophic retracting scar [74]. The scar did not regenerate by TAC injection after injury on the right foot. Therefore, the authors developed a new therapy using Om-PDRN and nucleotides. The Om-PDRN injection with nucleotides restored cutaneous texture in the dorsal and malleolar areas of the foot and improved pain and prediabetes. Additionally, the combined therapy resulted in clinically aesthetic and functional improvement without any treatment-related adverse events, thus allowing the patient to walk autonomously.

This review analyzes and summarizes the contents of related studies (Table 2, Table 3 and Table 4).

## 3. Methods

### 3.1. Search Strategy

We conducted a publication analysis on the biological effects of PDRN with the use of the keyword “polydeoxyribonucleotide” via Google Scholar, Pubmed, and Web of Science. We examined all language articles (609 articles) published from 2016 to 2020 except abstracts of symposia, patents, and bibliographic information. In this research, we searched to find in vitro, in vivo (animal model), and clinical articles of exclusive or combined usage of PDRN. We reviewed the in vitro, in vivo, and clinical articles on the biological effect of PDRN throughout the content of articles (in the abstract, methods and materials, results, and discussion sections).

### 3.2. Exclusion Criteria

We excluded 5 review articles on a specific disease to avoid repetitive article counting. However, we examined the content of articles that were mentioned in 5 review articles, and included them. Moreover, 534 studies simply described PDRNs as bioactive compounds in the introduction or discussion part. Therefore, we considered these 534 studies not relevant to the biological effect of PDRN and excluded them. We excluded four studies that did not mention extraction sources. Finally, we excluded one article that extracted PDRN from humans. Collectively, 544 studies were excluded in this review.

### 3.3. Inclusion Criteria

Five articles that contained both in vitro and in vivo studies were counted as two. Thus, seventy studies were analyzed to investigate trends in the research and development of PDRN. Among them, only one article applied PDRN using biomedical technologies as a hydrogel for diabetic wound healing.

## 4. Discussion and Conclusions

This review analyzed and discussed the therapeutic effects and applications of PDRNs extracted from marine organisms. A total of 70 studies published from 2016 to 2020 were analyzed and summarized. Analysis of the published years indicated that the highest number of PDRN-related articles during the study period was published in 2018, whereas 2019 had the lowest publication output. Nevertheless, publication numbers increased once again in 2020, suggesting that the number of studies on the therapeutic effects and applications of PDRNs will likely increase after 2020. As illustrated in Figure 3A,D, PDRNs primarily possess anti-inflammatory and wound healing properties, and are the mainly extracted from *O. keta* and *O. mykiss*.

Although PDRN exhibits many pharmacological properties, several limitations and weaknesses remain in terms of extraction source, used concentration, application, and underlying mechanism.

Firstly, *O. keta* and *O. mykiss* sperm is rather difficult to obtain, as these organisms only spawn during the breeding season, making Ok- and Om-PDRN prohibitively costly. Second, all studies in this review used commercially produced PDRN, not extract PDRN. Therefore, the concentration used in these experiments was fixed at under 1.875 mg/mL because commercial products are provided at a concentration of 5.625 mg/3 mL. Therefore, PDRN has the potential to show a stronger therapeutic effect at a higher concentration than 1.875 mg/mL. Regarding the modes of PDRN administration, most studies have injected these compounds directly at the wound site. Finally, the underlying mechanisms of PDRN were demonstrated by only five articles as MAPK activation [20,21,27,29,40]. Despite the limitations of PDRN, it has recently garnered much attention as a promising drug for several diseases.

Several sources other than *O. keta* and *O. mykiss* were used for PDRN extraction, including humans and plants. From 2016 to 2020, one article investigated the potential of PDRN extracted from humans on chronic ulcer treatment [78]. However, human PDRN has an ethic problem in obtaining an extraction source, similar to Ok- and Om-PDRN. Nowadays, several companies are trying to extract PDRN from plants such as roses, aloe, and broccoli [79,80,81]. When PDRN is extracted from plants, an additional process is required to break down the cell wall of plants, unlike animal-sourced PDRN. Due to the limitations of the alternatives (ethic problems and the need for additional processes), marine organisms are the best and most cost-effective materials from which to extract PDRN.

Biomedical engineering (BME) is a rapidly developing field of medical and biological research that provides new concepts and strategies for the diagnosis, treatment, and prevention of various diseases [82,83,84]. The main goal of BME is the development and application of new therapies and technologies to support, repair, or replace damaged cells, tissues, and organs with alternative systems of a bio-artificial nature [85]. Biomaterials are among the key elements of BME research and there are many ongoing efforts to develop more effective and safe biomaterials [86,87,88,89].

Therefore, additional research is needed not only to further characterize the therapeutic effects of PDRN but also to identify more cost-effective extraction sources such as starfish, algae, and unstudied marine organisms. Additionally, BME-based PDRN administration strategies, including hydrogels, three-dimensional scaffolds, nanofibers, and films, must be further developed to enhance the pharmacological activity of PDRN.

## Figures and Tables

**Table 1:** Other therapeutic effects of marine organism-derived PDRN.

Kind of Study	Source	Article Number	Therapeutic Effect
In vitro	*O. keta*	1	Alleviative effect on disease symptoms
		1	Biological effect (Anti-MMP, anti-melanogenesis, activating mitochondrial biogenesis)
		1	Dysfunctional tissue treatment
		1	OA treatment
	*O. mykiss*	1	Anti-melanogenesis
		1	OA treatment
In vivo	*O. keta*	1	Anti-osteonecrosis
		1	Anti-ulcer
		1	OA treatment
		3	Preventive effect on tissue damage
		1	RCTT treatment
		1	Tendon tear treatment
	*O. mykiss*	1	Anti-Allodynic
		1	Bone regeneration
		1	OA treatment
		2	RCTT treatment
	*A. sinensis*	1	Bone regeneration
		1	Preventive effect on tissue damage
Clinical	*O. keta*	1	Adjuvant therapy
		1	Alleviative effect on limb pain
		1	Alleviative effect on leg pain
		1	Alleviative effect on shoulder pain
		1	Anti-osteonecrosis
		1	Anti-ulcer
		2	CRPS treatment
		1	Preventive effect on PHL
		1	Preventive effect on scar forming
	*O. mykiss*	1	CRPS treatment
		1	Dysfunctional tissue treatment
		1	RCTT treatment
		2	OA treatment
		1	Preventive effect on PHL

**Table 2:** Effects of marine organism-derived PDRN through in vitro studies.

PublicationYear	Source	Stimulator	Cell	Outcomes	Target	Ref
2016	*O. keta* & *O. mykiss*	-	Mel-Ab & Co-culture of human melanocyte- keratinocyte model	↓ Synthesis of melanin ↓ Intracellular activity of tyrosinase ↓ Protein expression of MITF, tyrosinase, and TRP-1 ↑ Phosphorylation of ERK and AKT	Etc	[20]
2016	*O. keta*	-	U2OS &HDF	↑ Cell motilities	Wound healing	[8]
2017	*O. mykiss*	*E. coli* LPS (1& 10 ng/mL)	RAW 264.7	↓ Production of NO ↓ Secretion of IL-12 and TNF-α ↑ Secretion of IL-10 and VEGF-A	Inflammatory disease	[19]
2018	*O. keta*	IL-1β (10 ng/mL)	SW1353	↓ mRNA expression of CCL3, CCL4, CCL8, CXCL10, CXCL11, IL-1β, IL-6, IL-8, and TLR3 ↓ Protein level of CCL3, IL-1β, IL-6, and IL-8	Inflammatory disease	[17]
2018	*O. keta*	H_2_O_2_ (2 mM)	CHON-001	↓ Cell damage induced by H_2_O_2_ ↓ Expression of COX-2, PGE_2_ and TNF-α	Degenerative joint disease	[21]
2018	*O. keta*	ZA (10 μM) & *E. coli* LPS (0.1 μg/mL)	RAW 264.7	↑ Cell viability ↓ Production of NO ↓ Expression of IL-1β, IL-6, iNOS, and TNF-α ↑ Expression of VEGF and A_2A_R	Inflammatory disease	[18]
2018		IL-1β (10 ng/mL)	SW1353	↑ Protein expression of aggrecan, ANG-2, PDGF, and VEGF ↓ Protein expression of endostatin, angiostatin, and MMP13 ↑ Cell migration	Degenerative joint disease	[22]
2018	*O. keta*	-	CCD-986SK	↑ Phosphorylation of ERK, FAK, and JNK ↑ Cell migration	Wound healing	[27]
2019	*O. keta*	-	HGF (neonatal)	↑ Cell proliferation ↑ Expression of VEGF and CD31	Wound healing	[28]
2019	*O. keta*	*E. coli* LPS (1 μg/mL)	SH-SY5Y	↑ Cell proliferation ↓ Production and expression of IL-1β, IL-6, and TNF-α ↑ Phosphorylation of CREB and cAMP ↑ Expression of BDNF, cAMP and VEGF	Inflammatory disease	[25]
2020	*O. keta*	*E. coli* LPS (1 μg/mL) & TGF-β (5 ng/mL)	A549	↑ Cell viability reduced by LPS and TGF-β ↓ Expression of CTGF and hydroxyproline ↓ Expression of COL I, FGF, IL-6, and TNF-α	Etc	[24]
2020	*O. keta*	-	HDF & Diabetic HDF & HUVECs	↑ Cell proliferation and migration ↑ Expression of FGF and VEGF ↑ Vessel formation and density ↑ Total vessel network length	Wound healing	[8]
2020	*O. keta*	-	B16-F10 & CCD-986SK	↓ Activity of mushroom tyrosinase and the cellular tyrosinase ↓ Intracellular content and cellular level of melanin ↓ mRNA and protein expression of MITF, tyrosinase, TRP1, and TRP2 ↑ Mitochondrial density and the mtDNA contents ↓ Activity of in vitro collagenase and elastase ↓ Level of cellular MMP1 ↑ mRNA expression for mtDNA Antioxidant activity	Etc	[23]
2020	*O. keta*	PM10 (100 μg/mL)	NCI-H358	↓ Cytotoxicity induced by PM10 ↑ Concentration of cAMP ↓ Level of Caspase-3 and -9, IL-1β, IL-6, and TNF-α ↑ Phosphorylation of CREB and PKA ↓ Expression ration of Bax/Bcl-2 ↓ Expression of Cyt c and Apaf-1	Etc	[16]

↓: Decrease ↑: Increase.

**Table 3:** Effects of marine organism-derived PDRN through in vivo studies.

PublicationYear	Source	Treatment	Model	Outcomes	Target	Ref
2016	*O. mykiss*	Treatment of fractional ablative CO_2_ laser	Skin wounded SD rats	↑ Wound healing ↑ Epithelial confluence ↑ Score on granulation tissue thickness ↑ Number of VEGF-positive cells ↓ Erythema and crusting ↑ Number of PECAM-1/CD31-positive microvessels ↑ Production of VEGF	Wound healing	[46]
2016	*O. mykiss*	-	Subcutaneous implanted nude mice	↑ Number of osteoblast and fibroblast attached to the surface of each particle ↑ Area ratio of NB ↑ Number of bone-forming cells ↑ Deposition and calcification of NB matrix ↑ Development of the blood vessels Newly formed COL matrix Observed a fibrous capsule in vicinity of dentin particles	Etc	[36]
2016	*O. mykiss*	Intra-colonic injection of DNBS (25 mg) or Oral administration of DSS (8%)	Colitis-induced SD rats	↓ Colitis-induced damage ↓ Lipid peroxidation and neutrophil infiltration ↓ Serum level of IL-1β and TNF-α ↓ Bcl-2 expression ↑ Activation of A_2A_R	Inflammatory disease	[1]
2017	*O. mykiss*	CdCl_2_ challenge (2 mg/kg)	BTB integrity-induced C57 BL/6J mice	↓ Phosphorylation of ERK ↓ Level of FSH and LH ↑ Serum concentration of TE and inhibin B ↑ Size of seminiferous tubules ↑ Johnsen’s score ↑ Number of spermatozoa ↓ Isolation of peripheral positive germ cell and macrophages ↓Immunoreactivity on Claudin-11, N-Cadherin, occludin, and TGF-β3 ↓ Fragmented junctions between adjacent sertoli cells	Etc	[38]
2017	*O. mykiss*	Intra-tracheal instillation of *E. coli* LPS (5 mg/kg)	Lung injured SD rats	↓ Lung injury score and DNA fragmentation ↓ Expression of caspase-3, -8, and -9 ↑ Bax/Bcl-2 expression ratio ↑ Expression of A_2A_R, IL-6, and TNF-α Observed patch intra-alveolar macrophages Observed normal-looking alveolar structures except hyperplasia of type II pneumocytes	Inflammatory disease	[6]
2017	*O. mykiss*	-	Skin wounded SD rats	↓ Infiltration and number of inflammatory cells ↓ Scar size ↓ Expression of HMGB-1 ↑ Expression of COL I and III	Wound healing	[9]
2017	*A. sinensis*	-	Calvarial defected Wistar rats	↑ Percentage of NB ↑ Production of OCN and OPN	Etc	[13]
2017	*O. mykiss*	-	Tendon injured SD rats	↑ CSA of the laceration sites ↑ Resistant to mechanical stress ↑ Stored energy	Wound healing	[43]
2018	*O. keta*	Injection of MIA (60 mg/mL)	OA induced SD rats	↓ Expression of IL-1β, MMP-3, MMP-7 ↓ Phosphorylation of ERK ↓ Production of COX-2, PGE_2_, and TNF-α ↑ Regularity in chondrocyte distribution	Etc	[21]
2018	*O. mykiss*	-	Skin wounded ICR mice	↑ Wound healing↓ Infiltration of inflammatory cells ↑ Expression of p63, TGF-β, and VEGF ↑ Neovascularization Observed clear re-epithelialization and granulation tissue proliferation	Wound healing	[47]
2018	*O. keta*	-	Tendon injured New Zealand white rabbits	↑ Regeneration of COL fibers ↑ Number of VEGF-positive cells and density of PECAM-1-positive microvessel ↓ Tendon tear size	Etc	[5]
2018	*O. keta*	Oral administration of IND (20 mg/kg)	GU induced SD rats	↓ mRNA and protein expression of A_2A_R, IL-1β, IL-6, TNF-α, and VEGF ↑ cAMP concentration ↑ Phosphorylation of CREB and PKA Regenerated GU-induced tissue	Etc	[35]
2018	*A. sinensis*	CdCl_2_ challenge (2 mg/kg)	Brain damaged C57 BL/6J mice	↓ Level of MDA ↑ Level of GSH and BDNF ↓ Expression of mTOR ↓ Brain edema and ↓ Neuronal morphological changes	Etc	[14]
2018	*O. mykiss*	-	Tendon injured New Zealand white rabbits	↓ Mean tendon tear size ↑ Number of VEGF-positive cells and density of PECAM-1 positive microvessel ↑ Walking distance and fast walking time	Etc	[41]
2018	*O. keta*	-	Skin wounded hairless mice	↑ COL production ↓ Lipid accumulation Observed a normal wound healing process	Wound healing	[27]
2018	*O. keta*	Removal of ovary and uterus & Administration of ZA (0.6 mg/mL)	Osteoporosis inducedSD rats	↓ Severity of the osteonecrosis ↓ Necrotic bone formation at defect sites ↑ Number of blood vessels ↑ Number of attached osteoclasts ↓ Number of detached osteoclasts ↑ Recovery bone remodeling	Etc	[7]
2019	*O. keta*	Chondrocutaneous section of composite tissue	New Zealand White rabbits	↑ Average viable area ↑ Number of capillaries Observed the blood flow signal at the margin of the composite grafts	Wound healing	[45]
2019	*O. keta*		Skin wounded Cg-+Leprdb/+Leprdb & m+/+Leprdb mice	↑ Granulation tissue and capillary blood vessels ↓ Diabetic wound depth ↑ Proliferation of fibroblasts and ↑ Thickness of the epidermis ↑ Closure of diabetic wound ↑ Epithelialization of the epidermis	Wound healing	[28]
2019	*O. keta*	Devascularization of the descending colon	Ischemic colitisinduced SD rats	↓ Mucosal damage ↓ COL deposition in the colonic tissue ↓ Expression of A_2A_R, caspase-3, COX-2, IL-1β, IL-6, TNF- α and VEGF ↑ Expression ratio of Bcl-2/Bax ↑ Phosphorylation of ERK	Inflammatory disease	[29]
2020	*O. keta*	Oral administration of IND (20 mg/kg)	GU induced SD rats	↑ Mucosal tissue regeneration ↓ Histological score and ulcer index ↓ Expression and level of IL-1β, IL-6, and TNF-α ↑ Expression and level of cAMP expression ↓ Activation of NF-κB and MAPK pathway ↓ Expression ratio of Bax/Bcl-2 ↑ Activation of A_2A_R	Etc	[37]
2020	*O. keta*	Intratracheal instillation of *E. coli* LPS (5 mg/kg)	Lung injured SD rats	↓ Lung injury score ↓ Number of WBC and BALF ↓ Expression of, cleaved caspase-3 and -9, IL-1β, IL-6, TNF-α ↑ Expression of cAMP ↓ Expression ratio of Bax/Bcl-2 ↑ Phosphorylation of CREB and PKA ↓ Activation of NF-κB and MAPK pathways ↓ DNA fragmentation	Etc	[39]
2020	*O. mykiss*	SNL or CPIP	Neuropathic pain induced C57/Bl6 mice	↓ Mechanical allodynia ↓ Expression of GFAP	Etc	[33]
2020	*O. mykiss*	Injury on ACL	OA inducedWistar albino rats	↓ Total Mankin scores and structural integrity ↓ Cellular changes and tidemark continuity	Etc	[34]
2020	*O. mykiss*	Intra-colonic instillation of DNBS (25 mg/0.8 mL)	Colitis induced SD rats	↑ Colon length ↓ Abnormal condition of colon ↓ Extent and severity of colon injury and epithelial and mucosal alterations ↓ Ulceration, size of colitis, and hyperaemia ↑ Bcl-2 positivity ↓ Activity of MPO and lipid peroxidation	Inflammatory disease	[2]
2020	*O. keta*	I.p. injection of CCl_4_ (10 mL/kg)	Liver injured C57BL/6 mice	↓ Expression of CYP2E1 and UCP2 ↓ Gross morphology of the liver ↓ Liver weight, index, and histopathological score ↓ Concentration of TNF-α, IL-1 and IL-6 ↓ Activation of NF-κB and MAPK pathway ↓ Expression of cleaved caspase-3 and -9 ↓ Expression ratio of Bax/Bcl-2	Etc	[4]
2020	*O. keta*	-	Skin wounded C57BLKS/J-db/db mice	↑ Wound closure rate ↑ Thickness of the granulation tissue ↓ Number of inflammatory cells ↑ COL density of the regenerated tissue ↓ Expression of MPO and TGF-β ↑ Expression of VEGF and α-SMA	Wound healing	[8]
2020	*O. mykiss*	-	Tendon injured New Zealand white rabbits	↓ Mean STTS ↑ COL synthesis ↑ Density of PECAM-1-positive microvessel ↑ Fast walking time and walking distance and speed	Etc	[42]
2020	*O. mykiss*	I.p. injection of CCl_4_ (10 mL/kg)	Lung injured ICR mice	↓ AST and ALT concentrations ↑ Expression of A_2A_R ↓ Expression of IL-1β, IL-6, and TNF-α ↓ Expression ratio of Bax/Bcl-2 ↓ Percentage of TUNEL-positive cells	Wound healing	[40]
2020	*O. keta*	-	Tendon injured SD rats	↑ Tactile threshold for the von Frey filament test ↑ Paw withdrawal latency ↓ Concentration of IL-6 and TNF-α ↓ Number of cleaved caspase-3- and -9-positive cells ↑ Expression of cAMP ↑ Phosphorylation of CREB and PKA ↓ Expression ratio of Bax/Bcl-2	Etc	[44]

↓: Decrease, ↑: Increase.

**Table 4:** Effects of marine organism-derived PDRN through clinical studies.

PublicationYear	Source	Patient	Outcomes	Target	Ref
2016	*O. mykiss*	Patients with PHL (n = 8, Both)	↑ Number and thickness of hair ↓ Possibility of side effects	Etc	[69]
2016	*O. mykiss*	Patients with genital LS (n = 21, Male)	↑ Quality of life conditions of the patients No adverse reactions to the drug and no pain	Inflammatory disease	[48]
2016	*O. mykiss*	Patient with leg numbness (n = 1, Female)	↓ Allodynia and hyperalgesia ↓ Skin flushing Treatment of the acute inflammatory phase of CRPS type II	Etc	[66]
2016	*O. mykiss*	Patient with ankle pain (n = 1, Female)	↓ NRS score and pain No swelling and tenderness No complications	Etc	[70]
2017	*O. keta*	Patient with male PHL (n = 1, Male)	↑ Satisfaction of patient ↑ Hair counts, thickness, and graying	Etc	[68]
2017	*O. mykiss*	Patients with RCD (n = 106, Both)	↓ SPADI and VAS	Inflammatory disease	[49]
2017	*O. mykiss*	Patient with PA bursitis (n = 1, Female)	↓ NRS and pain No side effects	Inflammatory disease	[51]
2017	*O. keta*	Patient with neck and shoulder pain (n = 1, Female)	↑ Motor power of the left shoulder elevation and elbow flexion ↓ Pain of neck and left shoulder ↑ CMAP of the left musculocutaneous, axillary, and suprascapular nerve ↑ Expression of COL I and III, FGF, and VEGF Showed dense COL fibers with organized patterned arrangement	Wound healing	[72]
2017	*O. keta*	Patients with hemiplegic shoulder pain (n = 20, Both)	↓ Passive ROM and NRS	Etc	[64]
2017	*O. keta*	Patients with diabetic foot ulcer (n = 20, Both)	↓ Transcutaneous oxygen tension ↓ Inflammation and neutrophil pigmentation ↑ Granulation tissue formation	Etc	[55]
2017	*O. mykiss*	Patient with partial ear amputation (n = 1, Male)	↑ Survival of the composite ear graft Almost completely healed after 53 days	Wound healing	[75]
2018	*O. keta*	Patients with contracted nose (n = 30, Both)	↑ Mobility of nasal skin Softened the skin of contracted noses	Etc	[56]
2018	*O. mykiss*	Patients with chronic RCD (n = 32, Both)	↓ VAS score, pain, and SPADI ↑ Function and SANE No complications	Inflammatory disease	[50]
2018	*O. mykiss*	Patients with tendon tear (n = 17, Both)	↓ VAS at active shoulder ↓ DASH subscore and Global DASH ↑ ROM in forward flexion ↓ Tear volume of supraspinatus tendon No adverse events except one patient	Etc	[71]
2018	*O. keta*	Patients with lateral epicondylitis (n = 2, Male)	↓ Pain with decreased NRS ↓ Hypervascularity of common extensor tendon Improvement in the LE symptoms without any complications	Inflammatory disease	[52]
2018	*O. keta*	Patient with burn wound (n = 1, Male)	Improved adequately to wear the prosthesis after 4 weeks	Wound healing	[73]
2018	*O. keta*	Patients undergoing Thyroidectomy (n = 42, Both)	↓ VSS, EI, and height and width of scar ↓ Pigmentation and vascularity No adverse events	Etc	[57]
2018	*A. sinensis*	Patients with oral mucositis (n = 3, Both)	↓ Erythema, desquamation, and pain No allergic reactions	Inflammatory disease	[15]
2018	*O. keta*	Patient with CTS (n = 1, Female)	↑ NRS, BCTQ-severity and function scores No side effects	Etc	[61]
2018	*O. keta*	Patient with leg pain (n = 1, Male)	Pain relief	Etc	[65]
2018	*O. keta*	Patients with MRONJ (n = 5, Both)	Relief of pain and no sign of infection Observed soft tissue coverage of the operation area	Etc	[54]
2019	*O. mykiss*	Patients with knee joint pain (n = 29, Both)	↓ VAS, KSS, and WOMAC except stiffness No TEAEs, ADRs, and any other complications	Etc	[58]
2019	*O. keta*	Patient with limb pain (n = 1, Female)	↓ Left arm pain ↑ Motor strength	Etc	[60]
2019	*O. keta*	Patients with CTS (n = 30, Both)	↓ NRS, the severity score of BCTQ, and CSA	Etc	[62]
2020	*O. mykiss*	Patients with Knee OA (n = 98, Both)	↑ KSS	Etc	[59]
2020	*O. keta*	Patients with heel pain (n = 38, Both)	↓ VAS and MOXFQ scores No complications	Inflammatory disease	[53]
2020	*O. mykiss*	Patient with disabling scared (n = 1, Female)	↓ Pain and prediabetes Aesthetic and functional improvement Restored cutaneous texture Able to walk autonomously No TEAEs	Wound healing	[74]

↓: Decrease, ↑: Increase.

## Data Availability

Not applicable.

## Abbreviations

A_2A_R, Adenosine A2A receptor; ACL, Anterior cruciate ligament; ALT, Alanine aminotransferase; ANG-2, Angiopoietin-2; Apaf, Apoptotic protease activating factor; As-PDRN, PDRN extracted from *Acipenser sinensis*; AST, Aspartate aminotransferase; BALF, Bronchoalveolar lavage fluid; BCTQ, Boston Carpal Tunnel Syndrome Questionnaire; BDNF, Brain-derived neurotrophic factor; BTB, Blood-testis barrier; cAMP, Cyclic adenosine monophosphate; CCL, Chemokine (C-C motif) ligand; Cd, Cadmium; CD, Cluster of differentiation; COL, Collagen; COX-2, Cyclooxygenase-2; CPIP, Chronic post-ischemic pain; CREB, cAMP response element-binding protein; CRPS, Complex regional pain syndrome; CSA, Cross-sectional area; CTGF, Connective tissue growth factor; CTS, Carpal tunnel syndrome; CXCL, C-X-C motif chemokine ligand; Cyt c, Cytochrome c; DASH, Disabilities of arm, hand, shoulder; DNBS, Dinitrobenzenesulfonic acid; DRs, Adverse drug reactions; DSS, Dextran sulfate sodium; EI, Erythema index; ERK, Extracellular signal-regulated kinases; FAK, Focal adhesion kinase; FGF, Fibroblast growth factor; FSH, Follicle-stimulating hormone; GFAP, Glial fibrillary acidic protein; GHS, Glutathione; GU, Gastric ulcer; HA, Hyaluronic acid; HBOT, Hyperbaric oxygen therapy; HDF, Human dermal fibroblast; HMGB, High mobility group box; HUVEC, Human umbilical vein endothelial cells; i.p., Intraperitoneal; IL, Interleukin; IND, Indomethacin; iNOS, inducible nitric oxide synthase; JNK, c-Jun N-terminal kinases; KSS, Knee Society Scores; LH, Luteinizing hormone; LPS, lipopolysaccharide; LS, Lichen sclerosus; MAPK, Mitogen-activated protein kinase; MDA, Malondialdehyde; MIA, Monosodium iodoacetate; MITF, Microphthalmia-associated transcription factor; MMP, Matrix metalloproteinase; MOXFQ, Manchester-Oxford Foot Questionnaire; MPO, Myeloperoxidase; MRONJ, Medication-related osteonecrosis of the jaw; mtDNA, Mitochondrial DNA; NAS, Numeric rating scale; NB, New bone; NF-κB, Nuclear factor kappa-light-chain-enhancer of activated B cells; NO, Nitric oxide; NRS, Numeric rating scale; OA, Osteoarthritis; OCN, Osteocalcin; Ok-PDRN, PDRN extracted from *Oncorhycus keta*; Om-PDRN, PDRN extracted from *Oncorhycus mykiss*; OPN, Osteopontin; PA, Pes anserine; PDGF, Platelet-derived growth factor; PDRN, Polydeoxyribonucleotide; PECAM, Platelet endothelial cell adhesion molecule; PGE_2,_ Prostaglandin E_2_; PHL, Pattern hair loss; PKA, Protein kinase A; PM10, Particulate matter; PROM, Passive range of motion; PRP, Platelet-rich plasma; RCD, Rotator cuff disease; RCTT, Rotator cuff tendon tear; ROM, Range of motion; SANE, Single assessment numeric evaluation; SD, Sprague-Dawley; SNL, Spinal nerve ligation; SPADI, Shoulder pain and disability index; STTS, Subscapularis tendon tear size; TAC, Triamcinolone; TE, Testosterone; TEAEs, Treatment-emergent adverse events; TGF-β, Transforming growth factor-β; TLR, Toll-like receptor; TNF-α, Tumor necrosis factor-α; TRP, Tyrosinase-related protein; TUNEL, Terminal deoxynucleotidyl transferase dUTP nick end labeling; UCP2, Uncoupling Protein 2; US, Ultrasound; VAS, Visual analog scale; VEGF, Vascular endothelial growth factor; VSS, Vancouver scar scale; WBC, White blood cell; WOMAC, Western Ontario and McMaster Universities Osteoarthritis Index; ZA, Zoledronic acid; α-SMA, α-smooth muscle actin.
